# Supplementary material for: Modeling glioblastoma heterogeneity as a dynamic network of cell states
Source: Mol Syst Biol. 2021 Sep 16;17(9):e10105. doi: 10.15252/msb.202010105 (PMC8444284; doi:10.15252/msb.202010105)
Supplement: Supplementary file 6 — Source Data for Figure 5 [file MSB-17-e10105-s004.zip › Figure5A_sourcedata/GSEA_3071/nosigdb_stateI.GseaPreranked.1622795145664/NB_KI_CLUSTER18.html]

Details for gene set NB\_KI\_CLUSTER18[GSEA]

|  || Dataset | state13071 |
| Phenotype | NoPhenotypeAvailable |
| Upregulated in class | na\_pos |
| GeneSet | NB\_KI\_CLUSTER18 |
| Enrichment Score (ES) | 0.6910321 |
| Normalized Enrichment Score (NES) | 3.8525214 |
| Nominal p-value | 0.0 |
| FDR q-value | 0.0 |
| FWER p-Value | 0.0 |
Table: GSEA Results Summary

  

Fig 1: Enrichment plot: NB\_KI\_CLUSTER18      
 Profile of the Running ES Score & Positions of GeneSet Members on the Rank Ordered List

  

| PROBE | GENE SYMBOL | GENE\_TITLE | RANK IN GENE LIST | RANK METRIC SCORE | RUNNING ES | CORE ENRICHMENT || 1 | MGP |  |  | 1 | 2.652 | 0.0424 | Yes |
| 2 | COL3A1 |  |  | 3 | 2.646 | 0.0846 | Yes |
| 3 | COL1A1 |  |  | 6 | 2.008 | 0.1163 | Yes |
| 4 | PDGFRB |  |  | 9 | 1.893 | 0.1462 | Yes |
| 5 | COL6A1 |  |  | 10 | 1.797 | 0.1751 | Yes |
| 6 | FN1 |  |  | 11 | 1.769 | 0.2035 | Yes |
| 7 | COL6A3 |  |  | 13 | 1.717 | 0.2309 | Yes |
| 8 | TGFBI |  |  | 17 | 1.674 | 0.2569 | Yes |
| 9 | NUPR1 |  |  | 21 | 1.585 | 0.2815 | Yes |
| 10 | BGN |  |  | 24 | 1.568 | 0.3061 | Yes |
| 11 | COL4A1 |  |  | 28 | 1.524 | 0.3298 | Yes |
| 12 | IGFBP7 |  |  | 45 | 1.387 | 0.3474 | Yes |
| 13 | TIMP1 |  |  | 47 | 1.381 | 0.3693 | Yes |
| 14 | TFPI |  |  | 64 | 1.241 | 0.3847 | Yes |
| 15 | COL6A2 |  |  | 67 | 1.237 | 0.4040 | Yes |
| 16 | SERPINF1 |  |  | 71 | 1.211 | 0.4226 | Yes |
| 17 | COL4A2 |  |  | 73 | 1.203 | 0.4416 | Yes |
| 18 | ID3 |  |  | 86 | 1.152 | 0.4567 | Yes |
| 19 | THY1 |  |  | 94 | 1.114 | 0.4725 | Yes |
| 20 | ZFP36L1 |  |  | 97 | 1.096 | 0.4896 | Yes |
| 21 | NOTCH3 |  |  | 98 | 1.096 | 0.5072 | Yes |
| 22 | SPARC |  |  | 106 | 1.067 | 0.5223 | Yes |
| 23 | NEAT1 |  |  | 123 | 0.982 | 0.5335 | Yes |
| 24 | CST3 |  |  | 146 | 0.918 | 0.5418 | Yes |
| 25 | SERPINH1 |  |  | 157 | 0.902 | 0.5534 | Yes |
| 26 | ITGA1 |  |  | 160 | 0.891 | 0.5672 | Yes |
| 27 | IGFBP5 |  |  | 198 | 0.833 | 0.5698 | Yes |
| 28 | CALD1 |  |  | 201 | 0.824 | 0.5825 | Yes |
| 29 | RRBP1 |  |  | 208 | 0.810 | 0.5938 | Yes |
| 30 | MEF2C |  |  | 210 | 0.804 | 0.6064 | Yes |
| 31 | CEBPB |  |  | 222 | 0.782 | 0.6158 | Yes |
| 32 | LMNA |  |  | 247 | 0.739 | 0.6207 | Yes |
| 33 | IGFBP4 |  |  | 262 | 0.725 | 0.6283 | Yes |
| 34 | PRSS23 |  |  | 264 | 0.721 | 0.6396 | Yes |
| 35 | GSN |  |  | 265 | 0.720 | 0.6512 | Yes |
| 36 | IFITM3 |  |  | 268 | 0.714 | 0.6621 | Yes |
| 37 | UACA |  |  | 296 | 0.679 | 0.6652 | Yes |
| 38 | TPM2 |  |  | 320 | 0.655 | 0.6690 | Yes |
| 39 | COL18A1 |  |  | 336 | 0.638 | 0.6749 | Yes |
| 40 | ITGB1 |  |  | 371 | 0.615 | 0.6749 | Yes |
| 41 | FILIP1L |  |  | 387 | 0.602 | 0.6803 | Yes |
| 42 | LGALS3 |  |  | 390 | 0.600 | 0.6893 | Yes |
| 43 | MYL9 |  |  | 417 | 0.576 | 0.6910 | Yes |
| 44 | JUNB |  |  | 454 | 0.546 | 0.6894 | No |
| 45 | PDLIM1 |  |  | 539 | 0.487 | 0.6728 | No |
| 46 | CD63 |  |  | 618 | 0.444 | 0.6573 | No |
| 47 | CD99 |  |  | 659 | 0.430 | 0.6525 | No |
| 48 | MYH9 |  |  | 701 | 0.411 | 0.6472 | No |
| 49 | NR2F2 |  |  | 741 | 0.400 | 0.6423 | No |
| 50 | TIMP3 |  |  | 773 | 0.391 | 0.6396 | No |
| 51 | FOS |  |  | 832 | 0.370 | 0.6287 | No |
| 52 | CYTOR |  |  | 835 | 0.370 | 0.6341 | No |
| 53 | LY6E |  |  | 1030 | 0.321 | 0.5829 | No |
| 54 | VIM |  |  | 1105 | 0.305 | 0.5663 | No |
| 55 | SOCS3 |  |  | 1172 | 0.294 | 0.5519 | No |
| 56 | MYLK |  |  | 1204 | 0.287 | 0.5475 | No |
| 57 | PHLDA1 |  |  | 1262 | 0.275 | 0.5353 | No |
| 58 | CEBPD |  |  | 1310 | 0.267 | 0.5260 | No |
| 59 | CYR61 |  |  | 1318 | 0.265 | 0.5282 | No |
| 60 | CAV1 |  |  | 1341 | 0.261 | 0.5260 | No |
| 61 | SELENOM |  |  | 1365 | 0.258 | 0.5235 | No |
| 62 | A2M |  |  | 1808 | -0.295 | 0.3998 | No |
| 63 | FCGRT |  |  | 1871 | -0.301 | 0.3866 | No |
| 64 | CDKN1A |  |  | 2083 | -0.332 | 0.3307 | No |
| 65 | MFGE8 |  |  | 2468 | -0.404 | 0.2256 | No |
| 66 | ADAMTS1 |  |  | 2841 | -0.517 | 0.1258 | No |
| 67 | MT2A |  |  | 2910 | -0.547 | 0.1149 | No |
| 68 | RGS16 |  |  | 3134 | -0.708 | 0.0615 | No |
| 69 | LIMA1 |  |  | 3429 | -1.353 | -0.0022 | No |
| 70 | LHFPL6 |  |  | 3453 | -1.600 | 0.0169 | No |
Table: GSEA details [plain text format]

  

Fig 2: NB\_KI\_CLUSTER18: Random ES distribution      
 Gene set null distribution of ES for **NB\_KI\_CLUSTER18**
